# HDAC1 deregulation promotes neuronal loss and deficit of motor function in stroke pathogenesis

**DOI:** 10.1038/s41598-021-95837-3

**Published:** 2021-08-11

**Authors:** Jui-Sheng Chen, Hao-Kuang Wang, Chien-Yu Hsu, Yu-Ting Su, Jia-Shing Chen, Cheng-Loong Liang, Patrick Ching-Ho Hsieh, Cheng-Chun Wu, Aij-Lie Kwan

**Affiliations:** 1 Graduate Institute of Medicine, College of Medicine, Kaohsiung Medical University, Kaohsiung, Taiwan; 2 Department of Neurosurgery, E-Da Dachang Hospital, Kaohsiung, Taiwan; 3 Department of Neurosurgery, E-Da Hospital, Kaohsiung, Taiwan; 4 Institute of Biomedical Sciences, Academia Sinica, Taipei, Taiwan; 5 School of Medicine for International Students, College of Medicine, I-Shou University, Kaohsiung, Taiwan; 6 Department of Obstetrics and Gynecology, Kaohsiung Chang Gung Memorial Hospital and Chang Gung University College of Medicine, Kaohsiung, Taiwan; 7 School of Medicine, College of Medicine, I-Shou University, Kaohsiung, Taiwan; 8 Department of Neurosurgery, Kaohsiung Medical University Hospital, Kaohsiung, Taiwan

**Keywords:** Cell death in the nervous system, Diseases of the nervous system, Regeneration and repair in the nervous system

## Abstract

Stroke is a common cause of death worldwide and leads to disability and cognitive dysfunction. Ischemic stroke and hemorrhagic stroke are major categories of stroke, accounting for 68% and 32% of strokes, respectively. Each year, 15 million people experience stroke worldwide, and the stroke incidence is rising. Epigenetic modifications regulate gene transcription and play a major role in stroke. Accordingly, histone deacetylase 1 (HDAC1) participates in DNA damage repair and cell survival. However, the mechanisms underlying the role of HDAC1 in stroke pathogenesis are still controversial. Therefore, we investigated the role of HDAC1 in stroke by using a rat model of endothelin-1-induced brain ischemia. Our results revealed that HDAC1 was deregulated following stroke, and its expressional level and enzymatic activity were decreased. We also used MS-275 to inhibit HDAC1 function in rats exposed to ischemic insult. We found that HDAC1 inhibition promoted the infarct volume, neuronal loss, DNA damage, neuronal apoptosis after stroke, and levels of reactive oxygen species and inflammation cytokines. Additionally, HDAC1 inhibition deteriorated the behavioral outcomes of rats with ischemic insult. Overall, our findings demonstrate that HDAC1 participates in ischemic pathogenesis in the brain and possesses potential for use as a therapeutic target.

## Introduction

Stroke resulting from cerebral ischemia or hemorrhage is a common cause of neuronal cell death and neurological dysfunction in humans, leading to a cascade of events that include energy depletion and cell death (e.g., an excess of extracellular excitatory amino acids, free radical formation, and inflammation)^1,2^. Discovering therapeutics for improving the outcomes of ischemic brain injury remains challenging because ischemic damage engenders irreversible severe neuronal loss^3^. Consequently, an effective approach for preventing disease progression is still lacking.

Histone acetylation levels are determined by the equilibrium established between histone acetyltransferases and histone deacetylases (HDACs) that regulate transcription and other functions in the brain through chromatin conformation modulation. In general, HDACs function as transcriptional repressors to silence gene expression and promote chromatin compaction^4,5^. In the HDAC family, HDAC1 is involved in the transcriptional repression of cell cycle genes (e.g., E2F1, PCNA, and cyclins A and B). Aberrant cell cycle activity is involved in DNA damage under neurodegenerative conditions^6^. Notably, HDAC1 modulates DNA damage response through interaction with FUS^7^. In addition, HDAC1 plays an essential role in modulating neuronal death or survival during neurodegeneration; specifically, it interacts with HDAC3 to promote neuronal death and with histone-deacetylase-related proteins to promote neuronal survival^8^. Accordingly, HDAC1 is essential for neuronal survival.

In stroke, the role of HDAC1 remains controversial. Experimental cerebral ischemia^9^ as well as oxygen-glucose deprivation^10^ can induce HDAC1 upregulation, implying their involvement in ischemic pathology. In addition, pan-HDAC inhibitors significantly decrease neuronal injury and improve functional outcome in multiple preclinical models of focal ischemia^9,10^. By contrast, research reported that the levels of DNA transcription and protein translation of neuronal HDAC1 are downregulated following stroke^11^. In addition, HDAC1 downregulation was identified in oligodendrocytes after stroke^12^. Gain of HDAC1 function was demonstrated to have the potential to promote DNA damage repair and attenuate neuronal degeneration^6^. Because pan-HDAC inhibitor (pan-HDAC1i) toxicity affects diverse central nervous system cell types and pan-HDACi exhibits unexpected inhibitory effects in unique cell types^13,14^, identification of essential HDAC isoforms involved in stroke pathogenesis is required for developing therapeutic approaches for stroke.

In this study, we specifically examined the role of HDAC1 by using a rat model of endothelin-1 (ET-1) intracranial injection–induced brain ischemia^15,16^. HDAC1i MS-275 was used to dominantly inhibit the function of HDAC1 in order to elucidate the roles of HDAC1 in stroke pathogenesis^17^. We evaluated the levels and activity of HDAC1 after stroke as well as its involvement in DNA damage, neuronal apoptosis, reactive oxygen species (ROS), inflammation cytokines, and behavioral outcomes after brain ischemia. Our data indicated that HDAC1 is essential in stroke and is involved in DNA damage and cell apoptosis during ischemic pathogenesis, supporting HDAC1 as a specific target for developing stroke therapy.

## Results

### HDAC1 level and activity are deregulated after stroke

**Figure 1:**
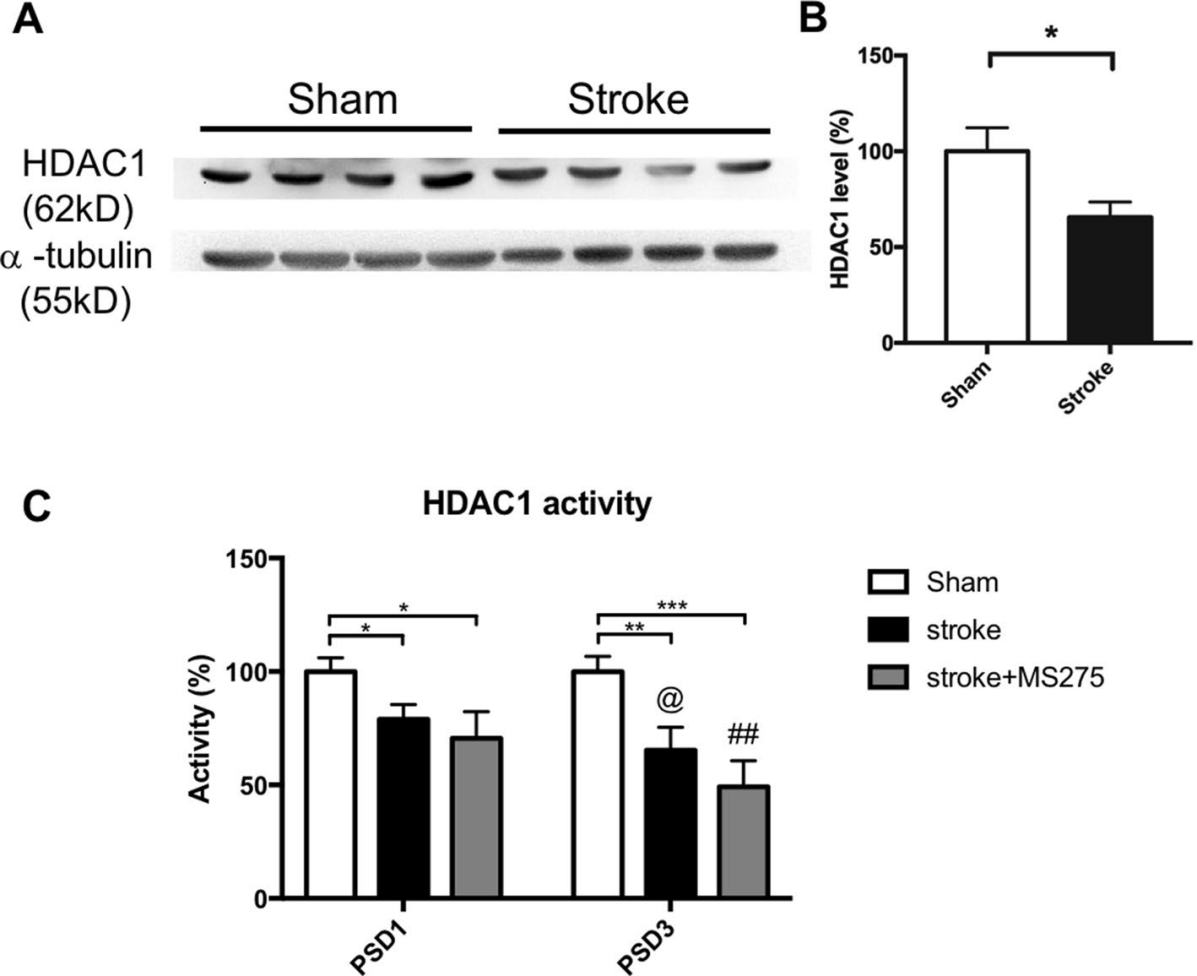
HDAC1 level and activity were deregulated after stroke. (**A**) Western blotting of HDAC1 expression in the ischemic brain region at 1 day after stroke. (**B**) Quantified result of HDAC1 from Western blotting. n = 8; *p < 0.05 by t-test. (**C**) The enzyme activity of HDAC1 at 1 day after stroke by immunoprecipitation of HDAC1 in brain lysates, and followed by conduction of enzyme activity assay for HDAC1. n = 8; ^@^denotes stroke PSD1 compares to PSD3, ^#^denotes stroke + MS275 PSD1 compares to PSD3. *Or one symbol denotes *p* < 0.005, **denotes *p* < 0.001, ***denotes *p* < 0.0001 by oneway-ANOVA.

To characterize the role of HDAC1 in ischemic pathogenesis, we first performed Western blotting to evaluate the expression levels of HDAC1 in the ischemic brain region on day 1 after stroke onset. The results revealed that the HDAC1 levels were reduced in rats with ischemic injury (Fig. 1A). Furthermore, the results indicated that compared with the sham brain, the ischemic brain exhibited lower levels of HDAC1 (Fig. 1B), suggesting that HDAC1 is involved in ischemic pathogenesis. Subsequently, to determine the enzyme activity of HDAC1 on day 1 and 3 after stroke onset, we immunoprecipitated HDAC1 in brain lysates obtained from the core and penumbra segmental region; we conducted enzyme activity assay for HDAC1 with equal HDAC1 concentration from the sham and stroke groups. In addition, to further elucidate the role of HDAC1 in ischemic damage, we used MS-275, a dominant HDAC1 inhibitor, to inhibit the function of HDAC1 following ischemic insult. MS-275 was synchronously injected with ET-1 by using a stereotactic apparatus. The result indicated that HDAC1 activity was repressed by ischemic insult (Fig. 1C); reduced HDAC1 activity was identified in the rat brain at 24 and 72 h after stroke. Furthermore, the data showed MS-275 significant repressed HDAC1 enzymatic activity (Fig. 1C) that further inhibited HDAC1 function in ischemic brain of rats at 24 and 72 h after stroke.

In addition, to evaluate whether MS-275 can induce self-toxicity in sham rats or the control group, we additionally conducted Triphenyl tetrazolium chloride (TTC) in sham rats with or without MS-275 intracerebral injection. Our data showed the data of infarct volume was no difference between both groups (Fig. S1A). Furthermore, we detected levels of lactate dehydrogenase (LDH) and reactive oxygen species (ROS) in the culture of primary neurons in vitro with or without MS-275 treatment and found no difference between both groups under normoxia conditions (Fig. S1B,C). These data suggest that MS-275 alone will not induce self-toxicity to injure neurons in vivo and in vitro, and support our animal model of HDAC1 inhibition is sufficient. Moreover, to understand the effect of MS-275 in HDAC1 level, we evaluated the level of HDAC1 at 24 and 72 h after stroke, the data showed stroke induced a lower HDAC1 level comparing to sham rats (Fig. S2), whereas MS-275 did not significantly reduce HDAC1 level in rats with stroke. (Fig. S2), suggesting MS-275 affects HDAC1 enzymatic activity but not protein level. Overall, our results indicate that HDAC1 was deregulated after stroke, implying its involvement in the pathogenesis of brain ischemia.

### HDAC1 inhibition promotes ischemic damage in stroke

**Figure 2:**
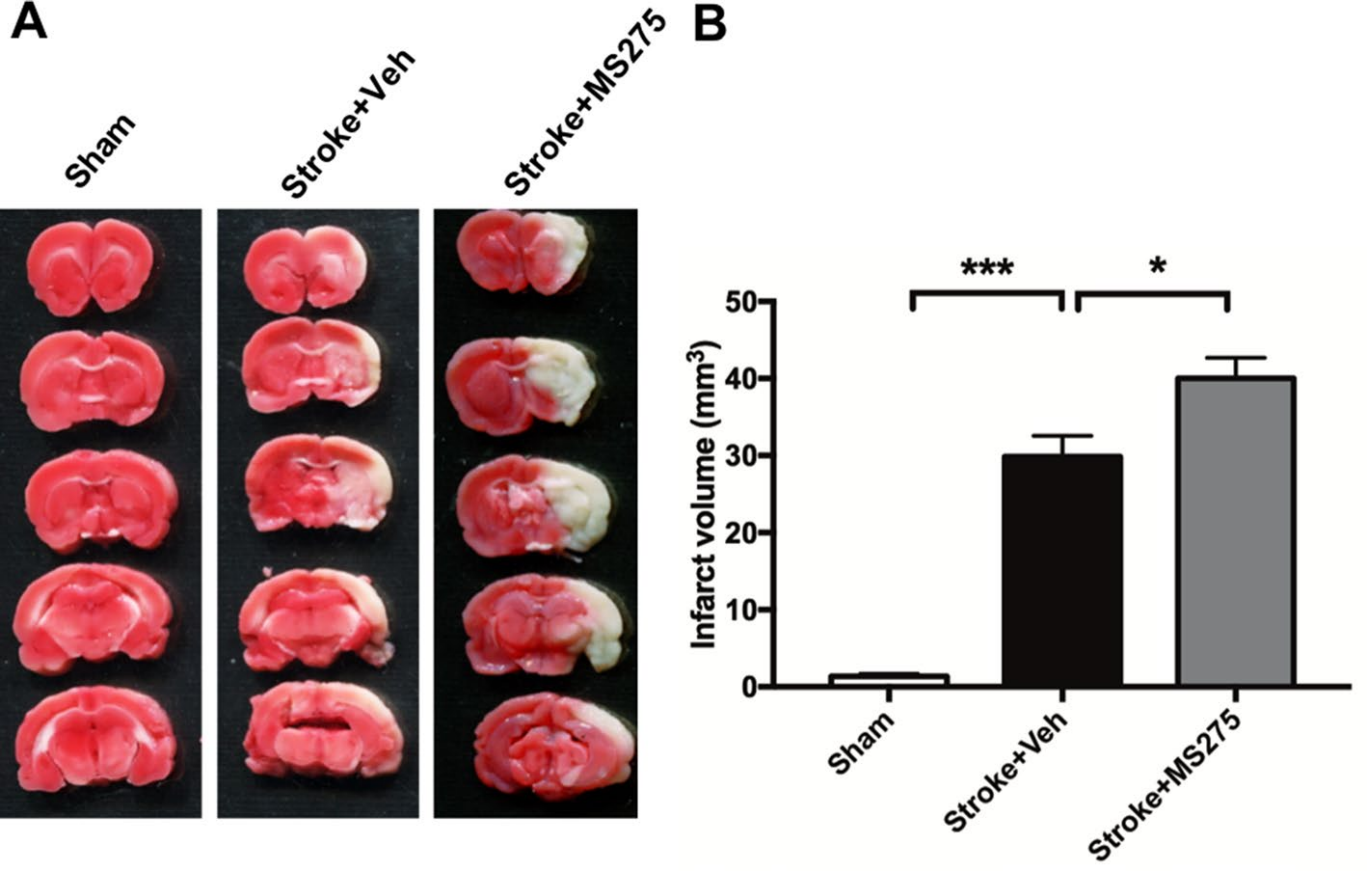
HDAC1 inhibition promoted ischemic damage in the brain after stroke. (**A**) The TTC staining of brain at 3 days after stroke with or without MS-275. (**B**) Quantification of infarct volume in rats with sham, stroke + vehicle or stroke + MS-275. n = 5; *p < 0.05, ***p < 0.001 by oneway-ANOVA.

To evaluate the effect of HDAC1 in stroke pathogenesis, we examined the infarct volume in our brain ischemic rat model. On day 3 after stroke onset, the rats were sacrificed and the brain removed for tetrazolium chloride (TTC) staining. Infarct regions were displayed in white, whereas unaffected regions were displayed in red (Fig. 2A). Compared with sham operation, ET-1 injection significantly induced ischemic damage within the cortex and striatum of the rat brain (Fig. 2A,B). In particular, rats that received the synchronous injection of ET-1 and MS-275 exhibited the largest infarct area. We also calculated the infarct volume and observed that it differed significantly between the brains obtained from rats injected with ET-1 along with or without MS-275 (Fig. 2B). These results reveal that HDAC1 dysfunction deteriorates ischemic damage and that HDAC1 has neuroprotective potential following ischemic insult.

### HDAC1 inhibition exacerbates neuronal loss in stroke

**Figure 3:**
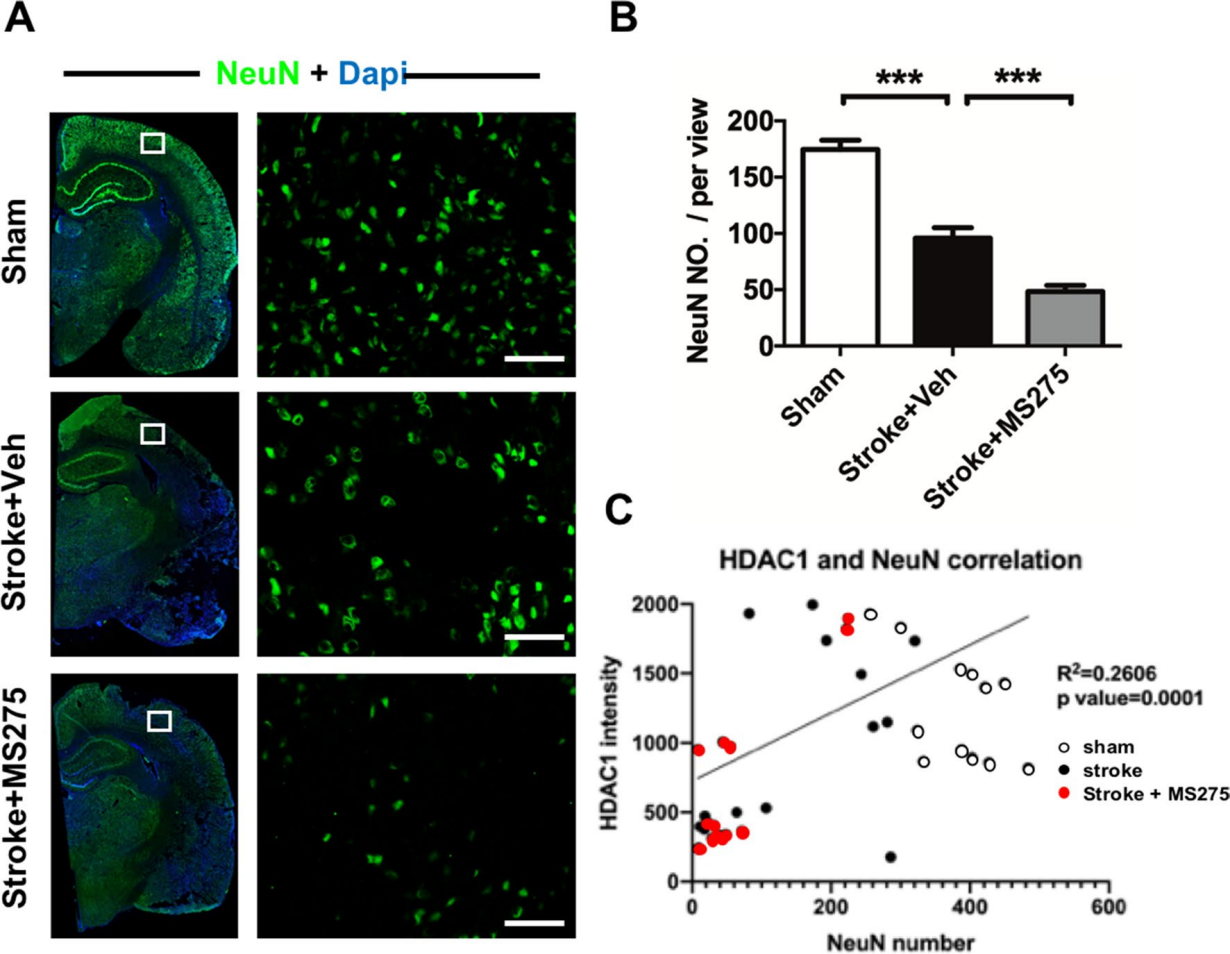
HDAC1 inhibition exacerbated neuronal loss in the brain after stroke. (**A**) Immunofluorescence staining of neuron cell (NeuN) with or without MS-275. The enclosed rectangle notes a sketch map of the areas where the images were acquired. (**B**) Quantification of the positive neuron cell number in the brain of rats with sham, stroke + vehicle or stroke + MS-275. n = 5 per group; ***p < 0.001 by oneway-ANOVA. (**C**) Correlation analysis for HDAC1 intensity and NeuN number. n = 5 per group. Bar: 100 μm.

To confirm whether HDAC1 dysfunction exacerbates neuronal loss after stroke, we examined the survival of neurons in the brain within ischemic core and penumbra regions on day 3 after stroke. Results obtained from immunofluorescent staining of NeuN indicated that ET-1 injection significantly reduced the neuronal number, and MS-275 injection further promoted ET-1-induced ischemic damage (Fig. 3A). Furthermore, rats that received the combined injection of ET-1 and MS-275 exhibited the most significant reduction in neuronal number after ischemic insult (Fig. 3B). In addition, We conducted immunofluorescent staining to co-label HDAC1 and NeuN as well as HDAC1 and γH2AX. Correlation analysis was used to evaluate the relation between HDAC1 expressional level and neuronal survival or DNA damage by quantification of HDAC1 immunoreactive density and NeuN or γH2AX positive numbers. The data showed that HDAC1 level was positive correlated with survived neuronal number (Fig. 3C. R^2^ = 0.2606, p = 0.0001). These data suggest that HADC1 dysfunction is essential for neuronal survival in stroke.

### HDAC1 inhibition is related to levels of DNA damage in stroke

**Figure 4:**
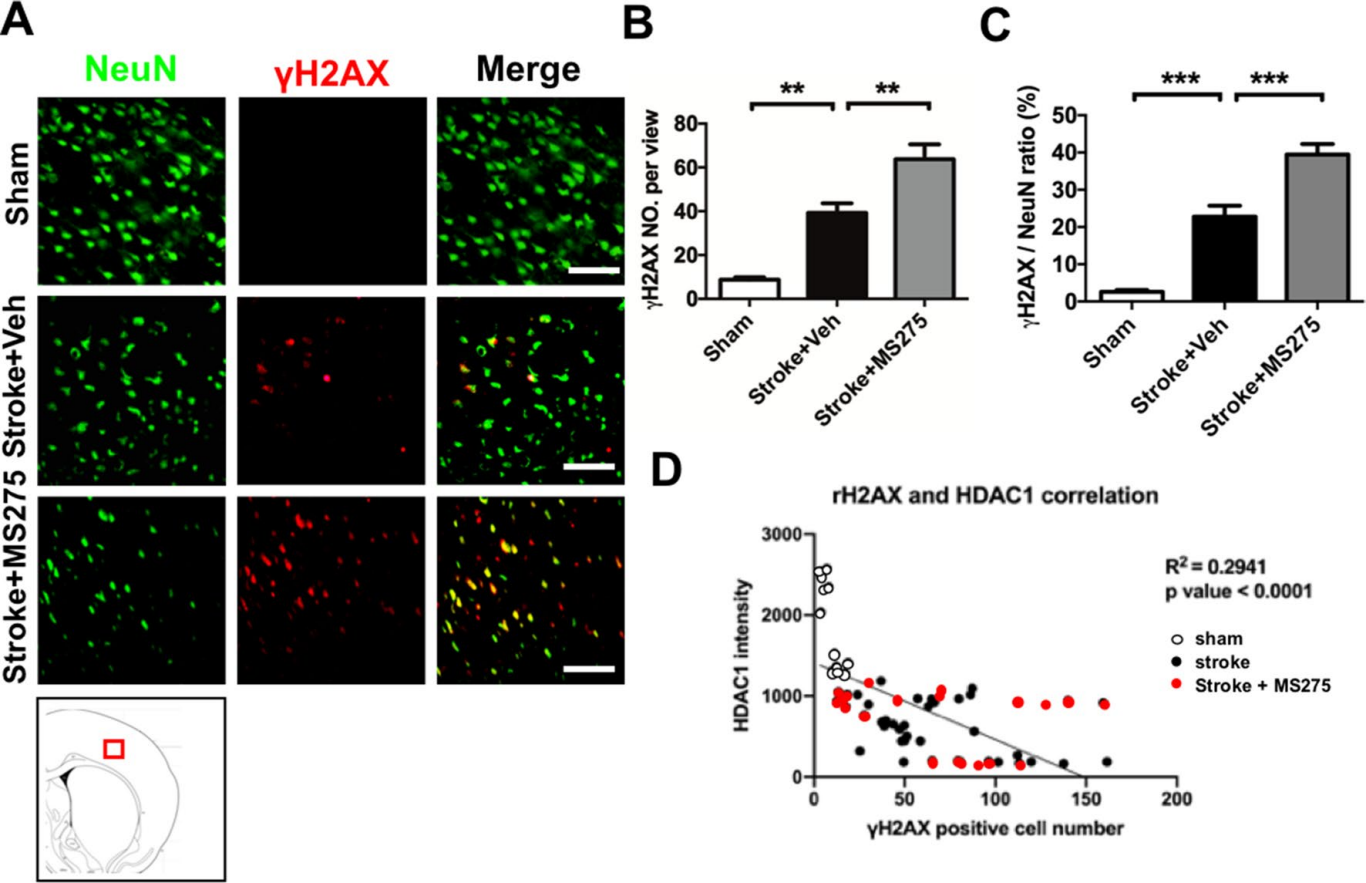
HDAC1 inhibition was related to levels of DNA damage in stroke. (**A**) Immunofluorescence staining of neuron cell (NeuN) and γH2AX with or without MS-275 respectively. A sketch map at the bottom notes the areas where the images were acquired. (**B,C**) Quantification of the number and ratio of γH2AX positive in neurons in the brain of rats with sham, stroke + vehicle or stroke + MS-275. n = 7; **p < 0.01, ***p < 0.01. (**D**) Correlation analysis for HDAC1 intensity and γH2AX cell number. n = 5 per group. Bar: 100 μm.

Excessive free radical species and reactive oxygen species may attack the DNA structure, resulting in DNA damage, which is a major cause of cell death in stroke. In stroke pathogenesis, expression of γH2AX reflects the presence of double-strand breaks in DNA^18^. Notably, HDAC1 was reported to be involved in the intracellular cascades of DNA damage response^19^. In the present study, to further determine the mechanism through which HDAC1 inhibition caused deterioration of neuronal loss, we evaluated the levels of DNA damage through immunofluorescent staining of NeurN and γH2AX in ischemic core and penumbra regions. The data revealed that ET-1 injection efficiently induced DNA damage on day 3 after stroke onset, MS-275 injection promoted this consequence, and rats that received both ET-1 and MS-275 injections exhibited the highest γH2AX immunoreactivity (Fig. 4A). Furthermore, we quantified the number of γH2AX-positive cells and ratio of γH2AX in the neurons, which revealed their significant roles (Fig. 4B,C). Moreover, in correlation analysis between HDAC1 level and γH2AX cell number, the data showed HDAC1 level was negative correlated with γH2AX positive cell number (R^2^ = 0.2941, p < 0.0001; Fig. 4D). These results thus demonstrate that HDAC1 inhibition could induce neuronal loss and is related to DNA damage.

### HDAC1 inhibition is related to levels of DNA fragmentation in rats with ischemia

**Figure 5:**
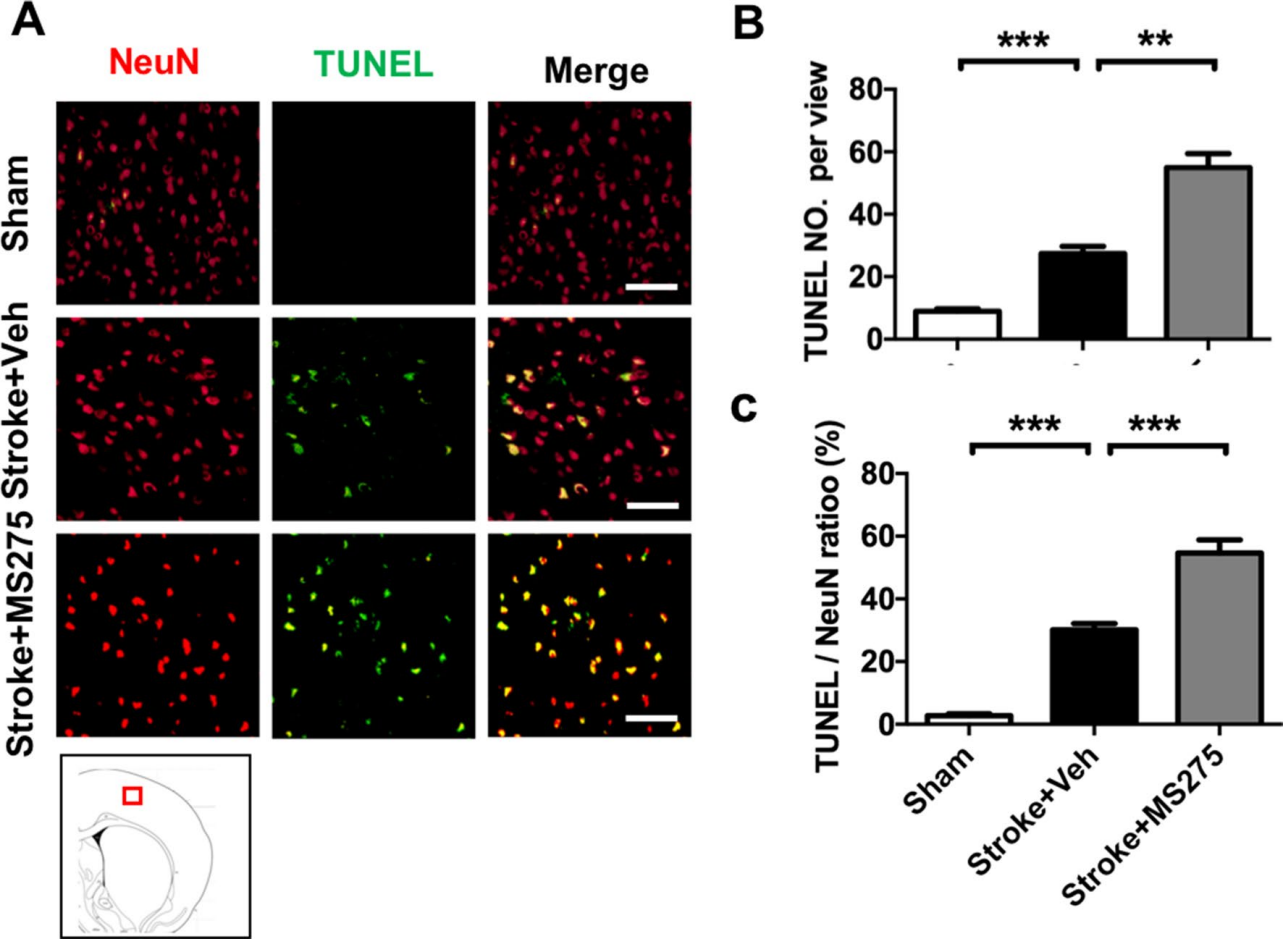
HDAC1 inhibition promoted cell apoptosis in the brain ischemia rats. (**A**) Immunofluorescence staining of neurons (NeuN) and TUNEL with or without MS-275. A sketch map at the bottom notes the areas where the images were acquired. (**B,C**) Quantification of the number and ratio of TUNEL positive in neurons in the brain of sham, stroke + vehicle or stroke + MS-275. n = 7; **p < 0.01, ***p < 0.01. Bar: 100 μm.

To confirm whether HDAC1 deregulation induced DNA damage in ischemic pathogenesis, we performed the terminal deoxynucleotidyl transferase dUTP nick end labeling (TUNEL) assay to detect the proportion of cells with DNA breaks in ischemic core and penumbra regions. Brain sections obtained on day 3 after stroke were counter stained with NeuN to validate neuronal loss. The results revealed that ischemia led to severe DNA damage in the rat brains and increased the number of TUNEL-positive cells (Fig. 5A). Furthermore, HDAC1 activity inhibition by MS-275 promoted ischemia-induced DNA damage, and more neurons with TUNEL immunoreactivity were identified in the brains of rats injected with MS-275 and ET-1. Moreover, the results revealed that the number and ratio of TUNEL-positive neurons increased in rats with ischemia, and HDAC1 inhibition further exacerbated the level of DNA damage in rats with ischemia (Fig. 5B,C), implying that HDAC1 inhibition can increase cell apoptosis.

### HDAC1 inhibition exacerbates levels of ROS, LDH, and inflammation

**Figure 6:**
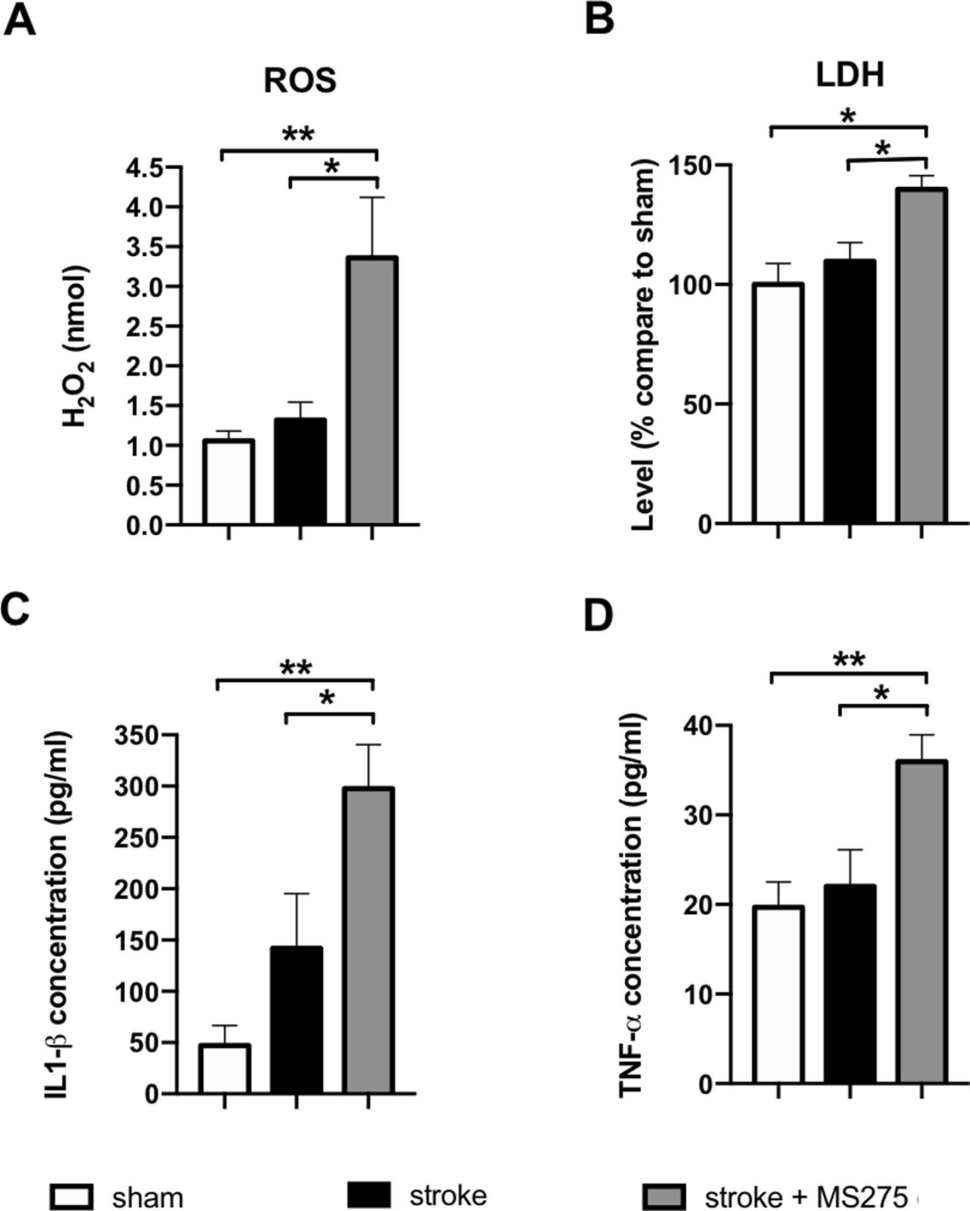
HDAC1 inhibition induced elevated levels of ROS, LDH, and inflammation cytokines. (**A**) ROS level and (**B**) LDH level assays were performed at 3 days after stroke in rats with sham, stroke + vehicle or stroke + MS-275. (**C**) Inflammation cytokine level of IL-1β and (**D**) TNF-α were examined at 3 days after stroke. n = 5; *p < 0.05, **p < 0.01.

In stroke pathogenesis, ROS and inflammation cytokines are known to contribute to secondary brain injury^20^. In particular, oxidative DNA damage is one of the most detrimental consequences of increased oxidative stress in brain ischemic insult^21^. To understand the effect of HDAC1 in pathologic mechanism of stroke, we examined the levels of ROS, LDH, IL-1β, and TNF-α on day 3 after stroke. The data indicated HDAC1 inhibition not only enhanced levels of ROS and LDH (Fig. 6A,B) but also promote synthesis of inflammation cytokines in IL-1β, and TNF-α (Fig. 6C,D). It suggests that HDAC1 is essential in secondary brain injury of stroke.

### HDAC1 inhibition reduces the outcomes of behavioral function in ischemic pathogenesis

**Figure 7:**
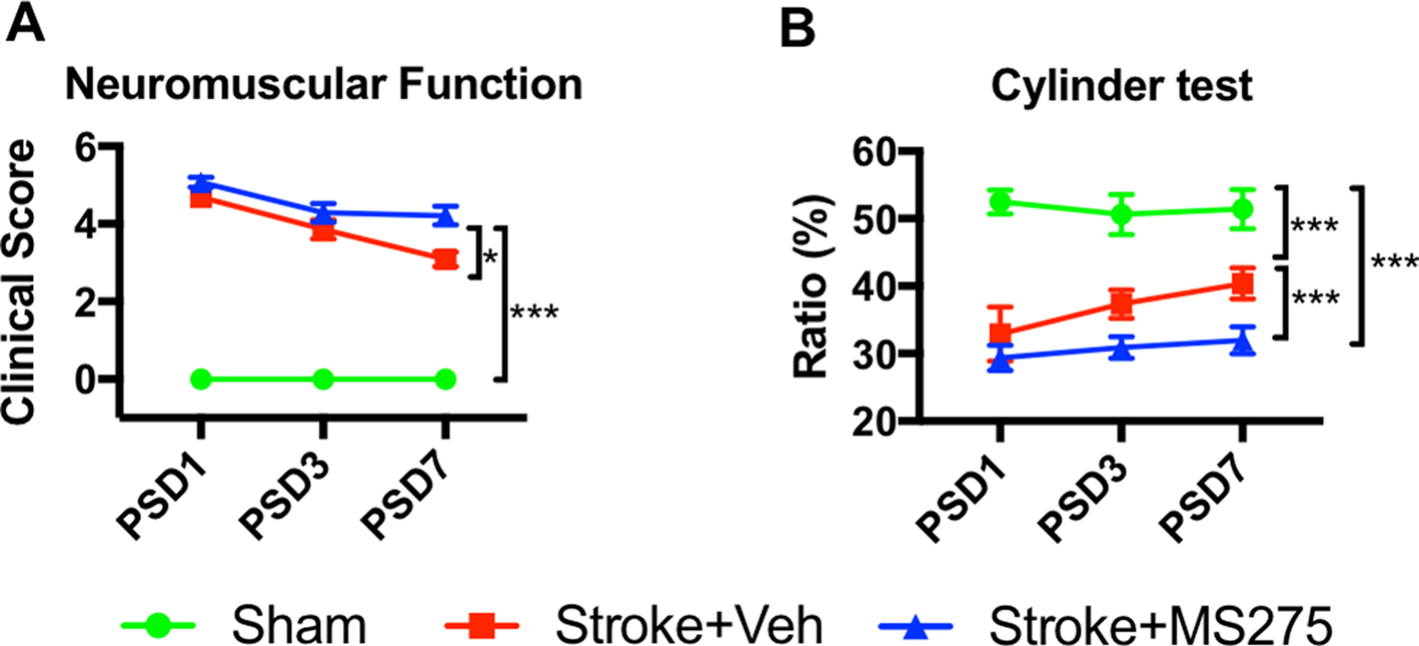
HDAC1 inhibition reduced the outcomes of behavioral function in the ischemia pathogenesis. (**A**) Neurological severity score test and (**B**) cylinder test were performed at 1, 3, 7 days after stroke in sham, stroke + vehicle or stroke + MS-275. n = 8; *p < 0.05, ***p < 0.001.

To determine the mechanism through which HDAC1 affects brain function in the pathogenesis of ischemia, we conducted a neurological severity score test and cylinder test to examine neurological function and spontaneous forelimb use. The results indicated that ischemic insult damaged neurological function in rats, and HDAC1 inhibition by MS-275 further deteriorated the outcome (Fig. 7A). In addition, the cylinder test indicated that rats with ischemic insult were worse in terms of the use of the forelimbs corresponding to the ischemic side; the rate at which such forelimbs were used also decreased. Notably, HDAC1 inhibition could aggravate this notion (Fig. 7B). These findings thus support that HDAC1 is involved in the modulation of behavioral function in rats with ischemia, which may result from HDAC1-deregulation-related neuronal damage and loss in the stroke pathogenesis.

## Discussion

In the pathogenesis of stroke, we found that HDAC1 levels and activity are reduced by ET-1. MS-275-induced HDAC1 inhibition promotes ischemic volume, neuronal loss, DNA damage, and neuronal apoptosis. Furthermore, our results indicate that HDAC1 inhibition deteriorates the behavioral outcomes of rats with ischemia due to neurological severity and motor function deterioration. These results demonstrate that HDAC1 participates in stroke pathogenesis that affects neuronal survival, DNA damage, apoptosis, and behavioral outcomes.

All HDAC family members present different functions in neurodegeneration (HDAC2 and HDAC3) and neuronal survival (HDAC5, HDAC7, and HDAC9)^22^. Notably, HDAC1 possesses both neurotoxic and neuroprotective roles with different cellular localization or interacting partners. HDAC1 mediates neurotoxic effect when exported from the nucleus to the cytoplasm for posttranslational modifications^23^. HDAC1 was reported to mediate the disruption of axonal transport and mitochondrial function in a multiple sclerosis mouse model^17^. In addition, HDAC1 was reported to interact with HDAC–related proteins, namely sirtuin 1 and FUS, for neuroprotective effects under normal conditions^7,24,25^. However, the role of HDAC1 in neurodegenerative diseases remains unclear.

The pathogenesis of ischemia brain damage is majorly characterized into early hyperacute (0–6 h), late hyperacute (6–24 h), acute (1 week), subacute (1–3 week), and chronic stages (more than 3 weeks)^26^. In acute stage, neuronal necrosis appears immediately following ischemia. The rest of neurons in penumbra region encounter other stress cascades, e.g., gliosis, inflammation, excitotoxicity, mitochondrial dysfunction, superoxide, DNA damage, BBB disruption, cell apoptosis…etc. in the following days^27^. These are critical events associated with neuronal survival. The neuronal loss starts from minutes after ischemia insult and the number is increased along the time course with a peak a 72 h after stroke and progressively decreased until about 1 week^28,29^. In addition, the data from clinical MRI also supports that scans with maximum diffusion-weighted imaging lesion volume occurred at a mean of 70.4 h^30^. Furthermore, inflammation is also a dominant threaten for neurons in penumbra region. Reactive microglia, astrocytes, and infiltrated monocytes are responded at about 24 h after stroke and progress to a peak at about 72 h after stroke, they mediated elevated cytokine level and superoxide accumulation induce secondary brain damage^27,29^. Accordingly, we selected PSD 3 and 7 as our observed time points.

In the application of MS-275, a concern was raised that whether MS-275 affected vasoconstrictive properties of ET-1. We found previous studies showed that HDAC1 modulates eNOS production as transcriptional repressor^31^ and endothelial morphogenesis is HDAC1 dependent^32^, implying that MS-275 potentially impairs vasoconstrictive properties of ET-1. However, our data from infarct volume and neuronal loss assessment excluded this speculation because MS-275 worsened the outcomes in rats with stroke. In addition, we further conducted primary neuron culture and oxygen/glucose derivation (OGD) to mimic ischemia/reperfusion in vitro. Our data showed MS-275 further promoted LDH and ROS production at 24 h after OGD (Fig. S3A,B) and MS-275 was selective to inhibit HDAC1 activity either in control or OGD conditions (Fig. S3C). These data suggest MS-275 did exacerbate neuronal damage by HDAC1 inhibition. Accordingly, the inferior consequence of infarct volume, neuronal loss, and behavior are dependent on neuronal damage but not the event affecting vasoconstrictive properties.

In the present study, we found that the inhibition of HDAC1 exacerbates neuronal loss and increases the infarct volume in stroke. Therefore, HDAC1 is neuroprotective in stroke pathogenesis. Furthermore, we observed increased γH2AX in the rat model of stroke. γH2AX acts as a marker in early cellular response to DNA double-strand breaks (DSBs) and is formed by H2AX phosphorylation by ataxia telangiectasia mutated kinase^33,34^. γH2AX formation relies on chromatin modification and initiates DNA repair responses^35^. Therefore, γH2AX acts as a marker of DNA DSBs. Previous studies have revealed that HDAC1 is recruited to DNA damage sites during DNA repair^36,37^. Moreover, HDAC1 interacts with sirtuin 1 and FUS, which mediate deacetylation of DNA repair proteins and other binding proteins^38,39^. The aforementioned evidence supports the relationship between HDAC1 and DNA repair. Accordingly, our findings further support the essential role of HDAC1 in stroke pathogenesis; HDAC1 inhibition aggravates neuronal loss and motor function deficits. The possible underlying mechanism is that HDAC1 mediates DNA repair.

We found that the MS-275 induced repression of HDAC1 enzymatic activity in vivo is not as significantly efficient as in vitro acquired data. We prospect it is due to the bioavailability limitation. However, the static significance in comparisons of groups and time points exists in Fig. 1C, we thus believe in the involvement of HDAC1 dysregulation in stroke pathogenesis. Additionally, MS-275 was stereotaxically injected into the brain via microinjection, this raised a question from penetration limitation of the injected reagents. Due to technical limitations, a maximum of 3 μL of reagent can be injected into the brain parenchyma, and the collected part of brain lysate may be much larger than the injected reagent can penetrate, this may further lead to the weak significance in Fig. 1C. Remarkably, in our previous reports, we conducted an HDAC1 specific activator named compound 5104434 in a transgenic neurodegenerative mouse model of frontotemporal lobe denegation^40^, our data indicated even the compound only trigger a minor increase of HDAC1, it is still sufficient to improve the behavioral outcomes in mice. It is reminiscent of the current study that a minor inhibition by MS-275 may induce exacerbation in the outcomes in rats after stroke.

Cell death in stroke is initiated through several mechanisms, such as inflammatory response, ATP depletion, calcium influx, and free radical release^41^, which can lead to motor, sensory, and cognitive function deficits. However, how HDAC1 regulates motor and sensory behaviors in stroke remains unclear. In this study, we used ET-1 to induce ischemic injury at sites adjacent to the surface of the middle cerebral artery area through an intracranial injection^15^. We subsequently recorded neurological severity scores and performed a cylinder test to evaluate neurological deficits^42^. The neurological severity score is a global neurological assessment, and the cylinder test is related to forelimb spatial and motor cortex function. We observed that the neurological severity score and cylinder behavior both deteriorated after HDAC1 dysfunction in stroke. The results suggest that HDAC1 participates in neuroprotection and affects behavioral outcomes in stroke.

In the evaluation of superoxide level, we aimed to understand the effect of HDAC1 in secondary brain injury. In early hyperacute stage, peroxynitrite is majorly resulted from ischemia neurons involving glutamate toxicity induced-nNOS production, dysfunction of mitochondria respiratory chain, and abnormal manifestation of NADPH oxidase^43^. Due to the short half-life of ROS, the ROS synthesized from early hyperacute stage is hard to be detected at 72 h after stroke. Therefore, we believe the signal from our data is based on inflammation and reactive glia-mediated ROS. In addition, our data showed HDAC1 inhibition promoted IL-1β and TNF-α levels. IL-1β is essential in pro-inflammatory and recruitment of neutrophil infiltration^44^. Neutrophils may further promote production of ROS causing oxidative stress^45,46^. TNF-α also participates in ROS production and promotes formation of infarct area in stroke pathogenesis^47^. Accordingly, we prospect that ROS level is resulted from reactive glia in secondary brain injury after stroke.

In this study, we determined that HDAC1 is deregulated in stroke pathogenesis. It participates in the modulation of ischemic volume, neuronal loss, DNA damage, cell apoptosis, and behavioral outcomes after ischemic insult. The findings of this study support that HDAC1 possesses potential for use as a therapeutic target.

## Material and methods

### Experimental animals and drug administration

The authors confirmed that all methods were carried out in accordance with relevant guidelines and regulations. A statement confirming the study was carried out in compliance with the ARRIVE guidelines. All experiments were conducted under the approval of the Institutional Animal Care and Use Committee (IACUC) at E-Da Hospital, Taiwan. Adult male Sprague–Dawley rats were purchased from Lasco biotechnology company (Taipei, Taiwan). The rats were used for all experiments when they weighted 250–300 g. Rats were allocated randomly to the following experimental groups: sham and ischemia insult with vehicle treatment, and ischemia insult with MS-275 treatment. To induce the ischemia stroke model, the vasodilator peptide intracranial injection was conducted. Briefly, 100 pM endothelin-1 (Sigma, E7764; St Lois, MO) reagent with or without MS-275 was stereotactic injected into the brain (AP 0, ML + 2.5, DV − 2.3; AP + 2.3, ML + 2.5, DV − 2.3; AP + 0.7, ML + 3.8, DV − 7.0)^48^. Endothelin-1 was dissolved in HBSS (Sigma, H6648). MS-275 was purchased from Sigma (EPS002). In our pilot study, we verified 100 μM of MS-275 was sufficient to induce worsened behavioral outcomes (Fig. S4), accordingly we adopted this dose in experimental assessments. In preparation for stereotaxic injection, 200 μM of MS-275 was pre-mixed with 200 pM of endothelin-1 as volume 1:1, and a total of 3 μL of mixed reagent was synchronized injected into the brain. For sham surgery, sham rats were stereotactic injected with HBSS only. If the rats did not appear behavioral deficits in modified neurological severity score test at 24 h after surgery, e.g., forelimb flexion, torso twisting, or abnormal manifestation in neurological responses, the rat will be excluded from the experiment. The mortality rate is about 10%, but no difference between stroke + vehicle and stroke + MS275 group.

### Western blot, ROS assay, LDH assay, ELISA

To analyze protein expression levels following ischemia insult and drug treatment, extracts were prepared from the brain tissue (bregma: + 3 to − 1 mm). To prepare the sample, we used a brain slicer to cut the brain into slices in the ischemic hemisphere. The detailed protocol for western blotting has been described previously^49^. The electrophoresis was conducted in 10% sodium dodecyl sulfate polyacrylamide gel, followed by blot hybridization with antibodies. The primary antibodies included HDAC1 (Millipore SAB1400120, Burlington, MA) and β-actin (Sigma A5316). The Image J software was used to quantify the relative intensities of the bands, which were normalized to the intensity of β-actin. ROS and LDH assay kits were buy from BioVision K936-100-250 and K726 (Millpitas, CA). ELISA kits for detection of IL-1β, and TNF-α were acquired from R&D RLB00 and RTA00 (Minneapolis, MN).

### HDAC1 activity assay

To detect the HDAC1 enzymatic activity, an activity assay kit was purchased from BioVision (Catalog# K342). Having the removed the brain, the nuclear protein extraction was conducted by an assay kit purchased from Millipore (Catalog# 2900) to isolate the nuclear protein, and then the nuclear protein was subjected to HDAC 1 immunoprecipitation for over night at 4 °C. Next day, the isolated HDAC1 was conducted the enzymatic activity assay followed the manufacture protocol.

### Infarct volume assessment

To quantify the ischemia infarct volume, 2,3,5-triphenyltetrazolium chloride (TTC; Sigma, T8877) was used. The procedures were conducted as previously reported^50^. Briefly, the rats were euthanized and the brains were removed, frozen, and coronally sectioned into 7 2-mm-thick slices using a rat brain slicer (World Precision Instruments, Sarasota, FL). The brain slices were incubated for 30 min in 0.2% TTC at 37 °C and fixed by immersion in a 4% paraformaldehyde solution. For each rat, data were collected from 6 TTC-stained brain sections. The images were analyzed by ImageJ (ver2.1, NIH). The unstained area of the brain sections was defined as the infarction area, and the margins were outlined manually. Total infarct volume was calculated and summarized from 5 brain sections.

### Immunofluorescent staining

The detailed protocol for IF staining was followed a previously paper^51^. To prepare the tissue for IF staining, the rats were anesthetized and perfused transcardially by PBS and 4% paraformaldehyde. The brain of each rat was removed and immersed in a 4% PFA solution for 2 h and dehydrated by gradient concentrations of sucrose. The sections were collected from bregma + 2 to − 4 mm; the cryo-tissues were sliced at 10 μm per section; one of three sections was collected and plated on the slide; 6 sections were collected on a slide, and 10 slides were prepared from a single rat brain. The penumbra region was identified by the intensity of immunoreactivity; the ischemic core exhibited only background from liquefactive necrosis. Twenty image views at least were quantified across 6 sections within penumbra regions of cortex and striatum. After preparing cryosections, the slides were incubated with a series of primary antibodies, including NeuN (Millipore, Catalog# MAB377) and γH2AX (Millipore, Catalog# 05636). The secondary antibodies used for the chromogenic reaction were AlexaFluor-conjugated secondary antibodies (Thermo, Waltham, MA). The sections were incubated with DAPI and mounted under coverslips with mounting medium (Dako, Glostrup, Denmark). The immunoreactive cells and positive area were quantified by ImageJ by setting threshold for the intensity of immunoreactivity^49^. In correlation analysis, immunofluorescent co-staining for HDAC1 and NeuN as well as HDAC1 and γH2AX were conducted. HDAC1 immunoreactive intensity and immune-positive cell number were quantified by ImageJ. Total 20 views from 5 rats per group were accumulated.

### Behavioral outcomes assessment

A focal scoring system for neurological severity score (NSS)^52^ was used to evaluate neurological outcomes of experimental rats on PSDs 1, 3, and 7. Three grade scores were designed to each animal, with functional measures including gait, body symmetry, climbing, turning behavior, fore limb extension, compulsory circling, and sensory response. Furthermore, cylinder test was conducted to evaluate forelimb deficits followed the previous paper^53^. The animal is placed in a transparent cylinder and evaluated. When assessing behavior in the cylinder, the number of independent wall placements observed for the right forelimb and left forelimb are recorded. The ratio of using ill site fore limb was quantified as R/(L + R) × 100%.

### Statistics

All data are presented as the mean ± SEM. All data were normal distributed and were analyzed by one-way ANOVA with post hoc Tukey’s test for multiple-group comparison or by Student’s *t*-test for 2-group comparison. Differences with *p* < 0.05 were considered statistically significant.

## Supplementary Information

Supplementary Information 1.

Supplementary Information 2.

Supplementary Information 3.

Supplementary Information 4.

Supplementary Information 5.
