# Supplementary material for: HDAC1 deregulation promotes neuronal loss and deficit of motor function in stroke pathogenesis
Source: Sci Rep. 2021 Aug 11;11:16354. doi: 10.1038/s41598-021-95837-3 (PMC8357973; doi:10.1038/s41598-021-95837-3)
Supplement: Supplementary file 1 — Supplementary Information 1. [file 41598_2021_95837_MOESM1_ESM.docx]

**HDAC1 Deregulation Promotes Neuronal Loss and Deficit of Motor Function in Stroke Pathogenesis**

Jui-Sheng Chen^1,2,3,4^, Hao-Kuang Wang^3,5^, Chien-Yu Hsu^3^, Yu-Ting Su^6^, Jia-Shing Chen^5^, Cheng-Loong Liang^3,7^, Patrick Ching-Ho Hsieh^4^, Cheng-Chun Wu^7#^, Aij-Lie Kwan^1,8*^

^1^Graduate Institute of Medicine, College of Medicine, Kaohsiung Medical University, Kaohsiung, Taiwan

^2^Department of Neurosurgery, E-Da Dachang Hospital, I-Shou University, Kaohsiung, Taiwan

^3^Department of Neurosurgery, E-DA Hospital, I-Shou University, Kaohsiung, Taiwan

^4^Institute of Biomedical Sciences, Academia Sinica, Taiwan

^5^School of Medicine for International Students, College of Medicine, I-Shou University, Kaohsiung, Taiwan

^6^Department of Obstetrics and Gynecology, Kaohsiung Chang Gung Memorial Hospital and Chang Gung University College of Medicine, Taiwan.

^7^School of Medicine, College of Medicine, I-Shou University, Taiwan

^8^Department of Neurosurgery, Kaohsiung Medical University Hospital, Kaohsiung, Taiwan

^#^ Co-corresponding author; ^*^ Corresponding author


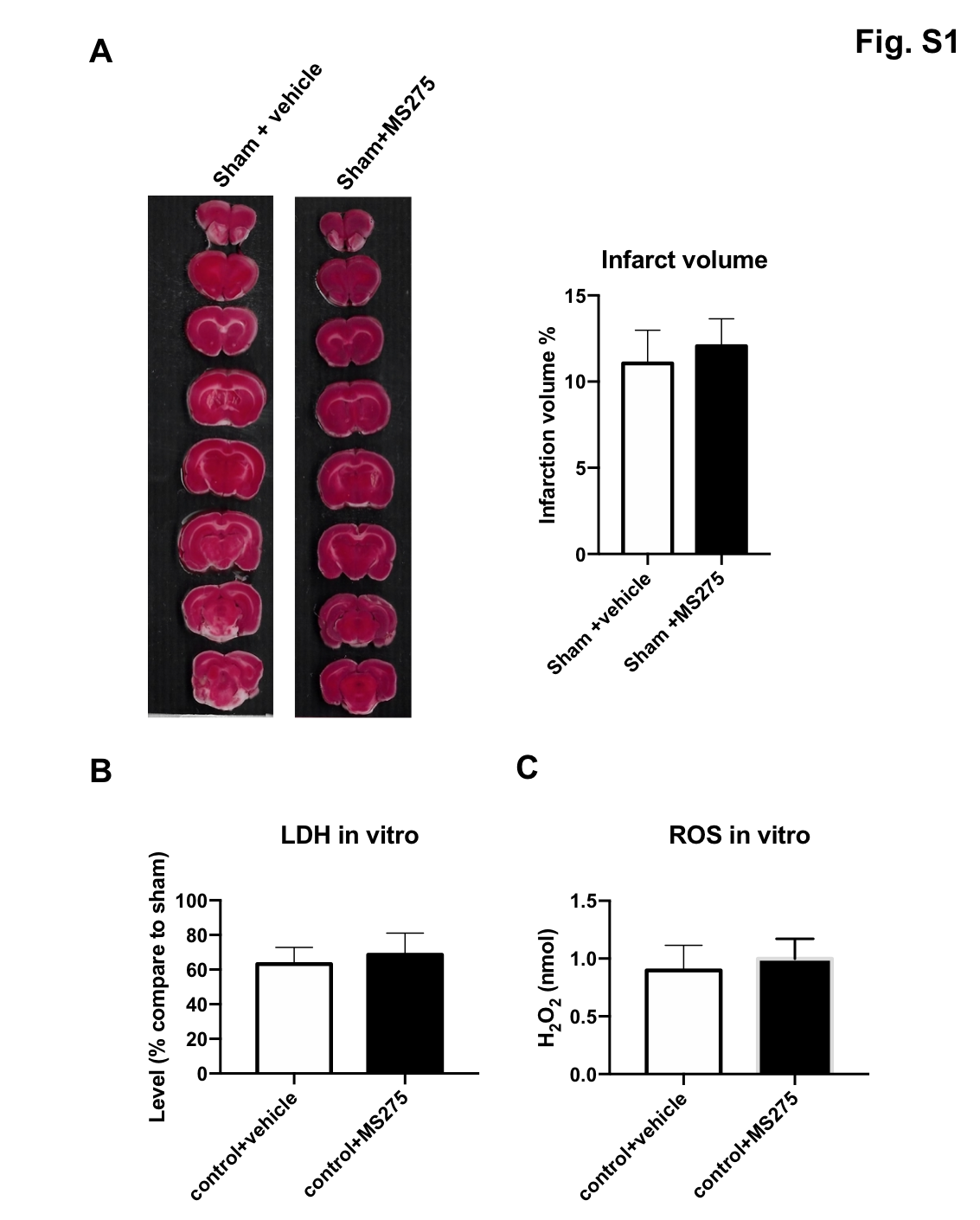


**Fig. S1. MS-275 treatment alone did not induce self-toxicity to damage neurons *in vivo* and *in vitro*.** (A) To evaluate whether MS-275 can induce self-toxicity in sham rats or the control group, we conducted triphenyl tetrazolium chloride (TTC) staining in sham rats with or without MS-275 intracerebral injection using the dose of 100 μM at 24 h after stroke. Our data showed the data of infarct volume was no difference between both groups. (B and C) We detected levels of lactate dehydrogenase (LDH) and reactive oxygen species (ROS) in the culture of primary neurons *in vitro* with or without MS-275 treatment (25μM) and found no difference between both groups under normoxia conditions.


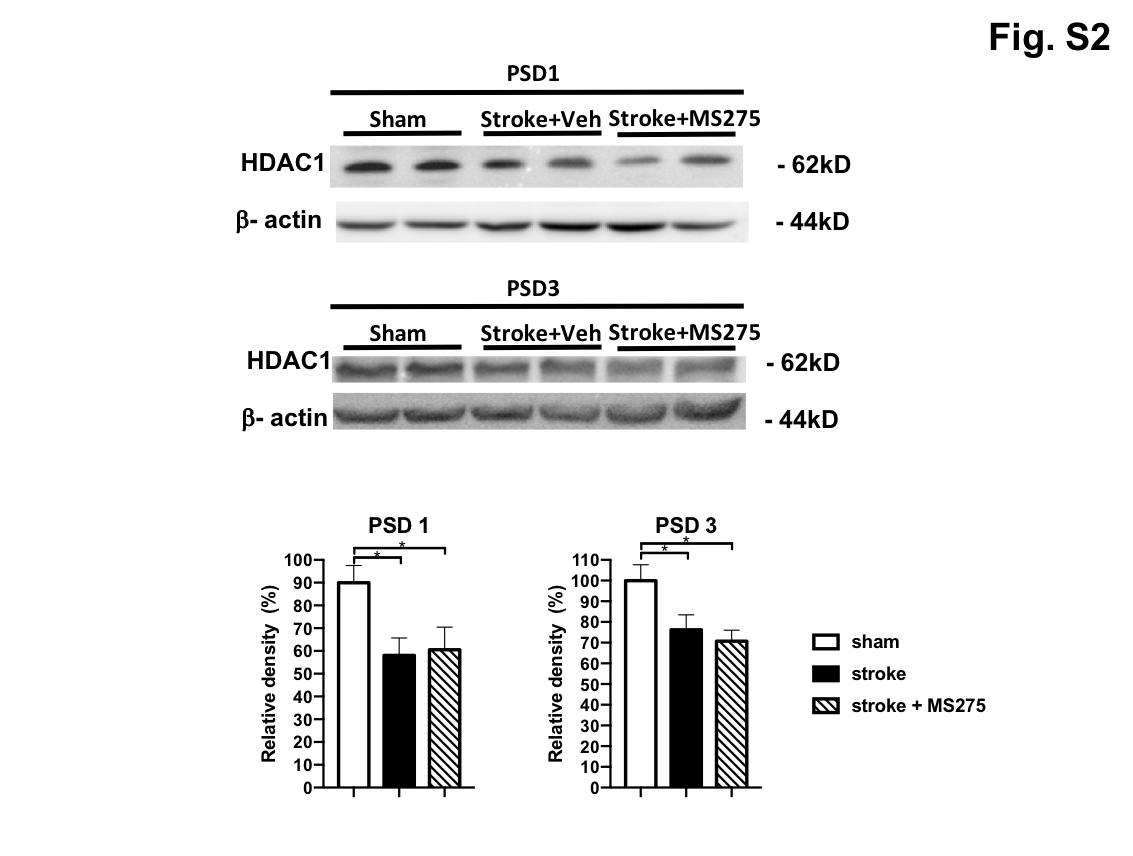


**Fig. S2. Endothelin-1-induced brain ischemia caused a decrease of HDAC1 level at PSD 1 and 3 after the surgery.** Western-blotting was adopted to evaluate the HDAC1 level at PSD 1 and 3 after stroke. The data showed ischemia/reperfusion significant repressed the level of HDAC1, whereas the MS-275 injection did not alter the HDAC1 level. n=4 per group, * denotes p< 0.05 by one-way ANOVA.


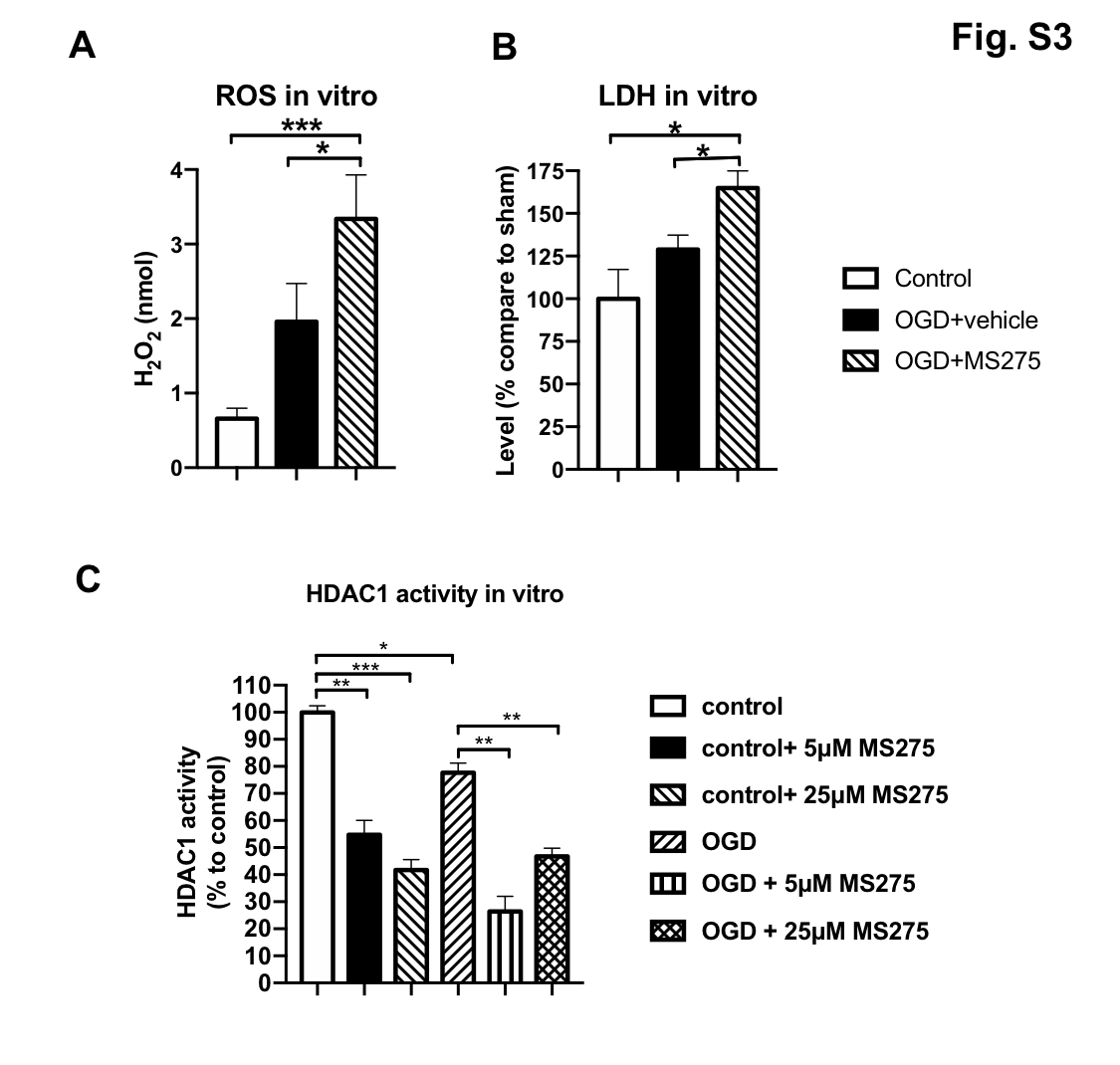


**Fig. S3. In vitro primary neuron based evaluations for effect of MS275 in levels of ROS, LDH, and HDAC1 activity.** Primary neurons were cultured for 9 days in vitro and subjected to oxygen and glucose derivation (OGD), the level of ROS (A), LDH (B), and HDAC1 activity (C) were examined. The data showed MS-275 administration in vitro exacerbated the levels of ROS and LDH production in neuron with OGD. MS-275 was selective inhibited HDAC1 activity either in control or OGD condition. n=3 independent experiments, * denotes p< 0.05, ** denotes p< 0.01,*** denotes p< 0.001 by one-way ANOVA.


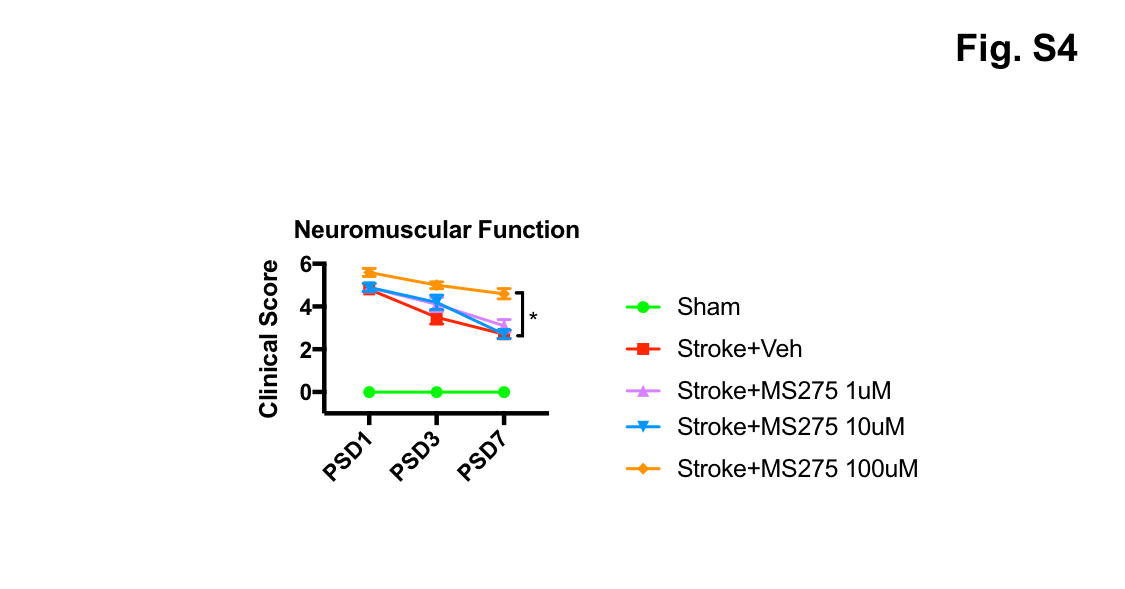


**Fig. S3. In vivo pilot studies for evaluation of MS275 doses in exacerbation of neuromuscular function.** Several doses of MS-275 were evaluated in 1, 10, and 100 μM in rats. The data indicated that the dose of 100 μM was sufficient to induce a worsened behavioral outcome at PSD 7. n=5 per group, * denotes p< 0.05, two-way ANOVA.
