# Supplementary figures and images for: HDAC1 deregulation promotes neuronal loss and deficit of motor function in stroke pathogenesis
Source: Sci Rep. 2021 Aug 11;11:16354. doi: 10.1038/s41598-021-95837-3 (PMC8357973; doi:10.1038/s41598-021-95837-3)

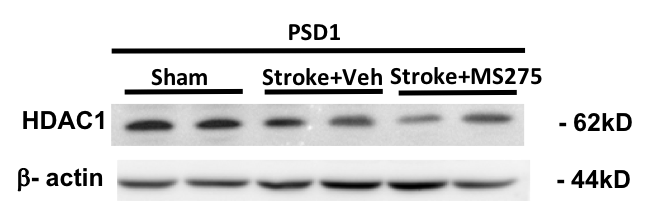

Supplement: Supplementary file 2 — Supplementary Information 2. [file 41598_2021_95837_MOESM2_ESM.png]

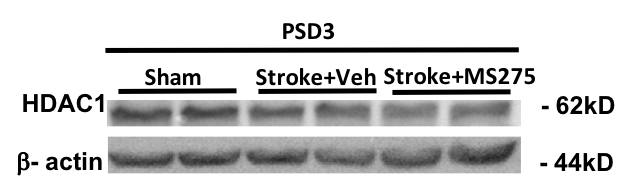

Supplement: Supplementary file 3 — Supplementary Information 3. [file 41598_2021_95837_MOESM3_ESM.png]

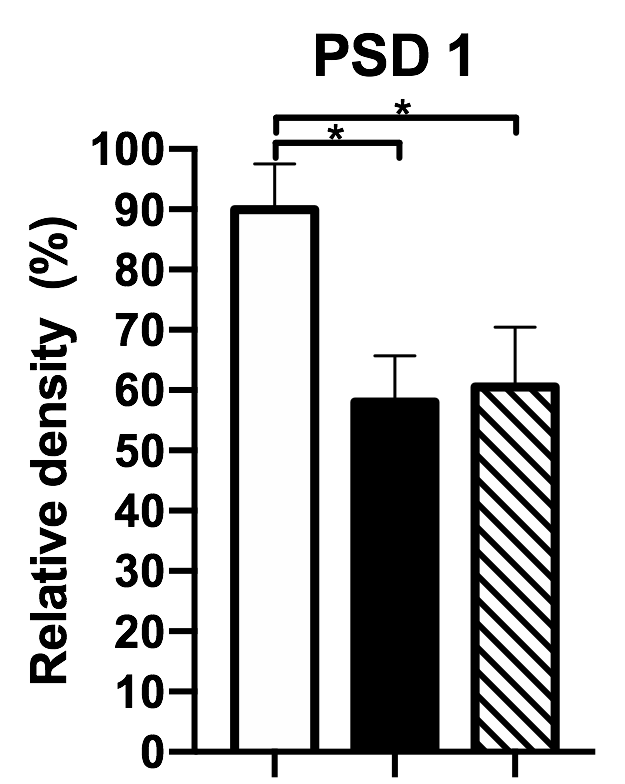

Supplement: Supplementary file 4 — Supplementary Information 4. [file 41598_2021_95837_MOESM4_ESM.tiff]

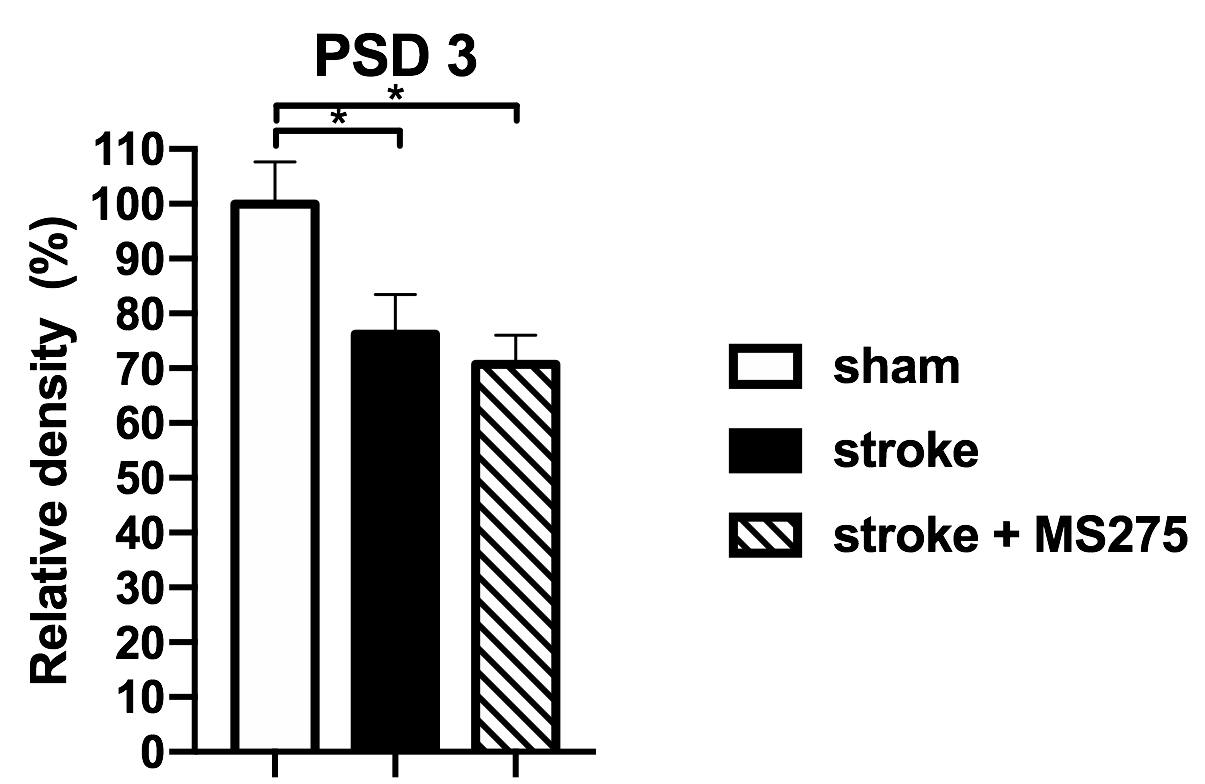

Supplement: Supplementary file 5 — Supplementary Information 5. [file 41598_2021_95837_MOESM5_ESM.tiff]
